# Plant-based Diet and Iron Deficiency Anemia in Sundanese Adolescent Girls at Islamic Boarding Schools in Indonesia

**DOI:** 10.1155/2021/6469883

**Published:** 2021-09-11

**Authors:** Mohammad Zen Rahfiludin, Septo Pawelas Arso, Tri Joko, Alfa Fairuz Asna, Retno Murwani, Lilik Hidayanti

**Affiliations:** ^1^Department of Public Health Nutrition, Faculty of Public Health, Diponegoro University, Semarang 50275, Indonesia; ^2^Department of Health Policy Administration, Faculty of Public Health, Diponegoro University, Semarang 50275, Indonesia; ^3^Department of Environmental Health, Faculty of Public Health, Diponegoro University, Semarang 50275, Indonesia; ^4^Department of Nutrition and Feed Science, Faculty of Animal and Agriculture, Diponegoro University, Semarang 50275, Indonesia; ^5^Department of Nutrition, Faculty of Health Science, Siliwangi University, Tasikmalaya 46115, Indonesia

## Abstract

**Background:**

Adolescent girls are at risk for iron deficiency anemia (IDA) due to the higher demand of iron for growth and the loss of blood during menstruation. Consumption of foods containing iron that have higher bioavailability can reduce the risk of IDA although diets that are largely plant-based, like those consumed by many Sundanese people, may not contain sufficient bioavailable iron. Here, we investigated the correlation between plant-based diets and IDA in adolescent Sundanese girls who were students at Islamic boarding schools in Indonesia.

**Methods:**

A total of 176 girls from seven Islamic boarding schools in Tasikmalaya were recruited. Nutritional intake data were obtained using 24-hr dietary recall. Blood samples were analyzed with a Sysmex-XNL instrument to measure several parameters including hemoglobin, mean corpuscular volume (MCV), mean corpuscular hemoglobin (MCH), and mean corpuscular hemoglobin concentration (MCHC).

**Results:**

The prevalence of IDA in the study population was 22.2%. Iron intake was 6.59 mg/day, which was lower than the recommended amount. The molar ratio of phytic acid to iron and vitamin C to iron was 8.72 and 0.03, respectively. There was a correlation between heme iron and both hemoglobin (*p*=0.009) and hematocrit (*p*=0.018). Iron from meat, fish, and poultry was correlated with hemoglobin (*p*=0.009) and hematocrit (*p*=0.011).

**Conclusion:**

The Sundanese plant-based diet did not affect the IDA status. Instead, IDA was associated with consumption of less animal-based foods that have iron with higher bioavailability. Increased access to an animal-based menu at the school cafeteria could be an approach to prevent IDA in students at Islamic boarding schools in Indonesia.

## 1. Introduction

Adolescence spans the ages between 10 and 19 years and is a period of marked physical growth. During this stage, adequate nutritional status of females, in particular, can not only determine the current quality of life but also indirectly affect the nutritional status of future children and the ability to care for and nourish them adequately [1]. In our previous study, we found that more than half of pregnant mothers in the study population were anemic (50.7%) and iron deficient (69.6%), indicating that appropriate measures were urgently needed to avoid health issues associated with anemia and iron deficiency [2].

An increase in lean body mass, blood volume, and red cell mass during the rapid growth in adolescence can deplete iron stores and increase the risk of iron deficiency [3]. In low- and middle-income countries, many adolescents have iron deficiency anemia (IDA) that results from malnutrition during childhood [4]. The prevalence of iron deficiency and IDA is higher among adolescent girls than boys [5] in part due to blood loss during menstruation in addition to increased nutritional requirements for growth [6].

Higher demand for iron requires increased consumption of iron-rich foods. Dietary iron exists as heme and nonheme iron. Heme iron, which is mainly found in meat, fish, and poultry, has better bioavailability than nonheme iron, which is found mostly in plant-based foods [7]. Although heme iron is estimated to contribute 10%–15% of total iron intake in meat-eating populations due to its higher and more uniform absorption (estimated at 15%–35%), heme iron could contribute ≥40% of total absorbed iron. Meanwhile, nonheme iron constitutes a greater portion of the total iron in foods, but its absorption is low and is affected by the presence of soluble enhancers and inhibitors consumed before or with the meal [7, 8].

In plant-based diets, phytate is the main inhibitor of iron absorption [8]. Phytate occurs when phytic acid, a negatively charged molecule, binds to mono‐ and divalent dietary mineral cations to form highly stable phytate complexes at neutral pH. As a divalent cation, iron bioavailability in the gastrointestinal tract decreases, and the small intestine pH increases the dissociation and formation of phytate‐divalent cation complexes that precipitate, thus lowering availability for absorption [9]. Polyphenols widely present in coffee and tea can also strongly inhibit dietary nonheme iron absorption. Consumption of a cup of tea with a meal decreased iron absorption by 59% in people with IDA and 49% in healthy individuals [10]. In contrast, the presence of vitamin C (both synthetic and dietary) is the most significant enhancer of iron absorption and can enhance iron absorption up to 6 fold in those who have low iron stores, thus overcoming the inhibitory effects of phytic acid. Vitamin C facilitates nonheme iron absorption by reducing ferric (Fe^3+^) to ferrous (Fe^2+^) iron, which is more easily absorbed [11].

The nutritional adequacy of iron in food is determined by the amount and quality of iron in the food consumed. The quality of iron is affected by its bioavailability, which is expressed as the proportion of iron consumed compared to iron that is absorbed and used for physiological functions. Iron storage is influenced by food and physical condition [12]. The bioavailability of iron can be estimated by calculating the molar ratio in the diet. A molar ratio of phytic acid to iron >1 indicates inhibition of iron absorption [13]. Meanwhile, consumption of vitamin C having a molar ratio of 2 : 1 can overcome the inhibition of iron absorption caused by phytic acid [14].

As an excellent source of iron, meat is an important factor that must be considered for prevention of IDA. However, meat consumption in developing countries remains low. In Indonesia, per capita beef consumption is only 2.7 kg per year, which is low compared to other countries in Southeast Asia, such as Malaysia (15 kg) and the Philippines (7 kg), and particularly low compared to other large countries such as Australia (90.2 kg), the United States (90 kg), Argentina (86.5 kg), and Brazil (78 kg). Meanwhile, chicken and fish consumption in Indonesia is higher with per capita consumption around 15 kg and 32.4 kg per capita per year, respectively [15].

Indonesian diets are still dominated by plant-based foods [16]. Vegetable consumption in Indonesia in 2018 was approximately 54 kg per capita per year [17], which was higher than other Asian countries such as Malaysia (46.9 kg), Thailand (37.6 kg), Sri Lanka (31.6 kg), and Bangladesh (20.5 kg) [18].

As the largest archipelagic country in the world, the territory of Indonesia consists of 17 thousand islands inhabited by more than a thousand ethnicities with different food cultures. Up to 15.5% of the population in Indonesia is Sundanese, which is the second largest ethnic group in Indonesia. Many Sundanese people originate from West Java [19]. Sundanese food includes many vegetables, such as karedok (raw vegetable salad in peanut sauce), lalapan (a variety of raw vegetables served with chili sauce), and sayur asem (vegetable tamarind soup). Hence, we examined whether the plant-based dietary habits of Sundanese in Indonesia significantly affect their health. We also assessed the correlation between iron consumption from a plant-based diet by considering the molar ratio of vitamin C and phytic acid to iron with the iron status in Sundanese adolescent girls studying at Islamic boarding schools in Indonesia.

## 2. Materials and Methods

### 2.1. Study Design and Subject

This was a quantitative study with an analytical design and a cross-sectional approach. The subjects were female students who were randomly selected from seven Islamic boarding schools in Tasikmalaya, West Java province, Indonesia. The sample size was calculated based on the formula for the minimum number of subjects for a cross-sectional study. Based on a previous local study that estimated a prevalence of anemia among junior high school students of 50%, the minimum sample size for this study was 171 adolescent girls. A total of 176 were enrolled to account for the possibility of nonresponse [20]. Female students who were both living and eating at the school who were willing to participate and provide informed consent were enrolled in the study.

### 2.2. Measurements

Data on subject characteristics were obtained through face-to-face interviews with subjects. Nutritional intake data were obtained using a 24-hour dietary recall method for three nonconsecutive days. Dietary patterns for sources of iron and enhancers and inhibitors of iron absorption were also assessed. Although students lived in the school dormitory and were provided three meals a day, some students bought food outside the school compound. Thus, we recorded the daily meal the students received at the school cafeteria and also food or snacks bought outside of school. Food intake was recorded in the form of household portions (tablespoons, teaspoons, cups, etc.). Pictures were used as a visual aid to determine portions consumed. Food intake was converted into grams and analyzed using the Nutrisoft software to calculate nutritional intake. All nutritional intake (except phytic acid) was categorized into two groups, inadequate and adequate, according to the recommended dietary allowance (RDA) in Indonesia. Phytic acid intake was classified as inadequate if the amount consumed was ≤650 mg/day [21].

The moles of phytic acid, vitamin C, and iron were determined by dividing the weight per 100 grams of food by the atomic weight (phytic acid: 660 g/mol; vitamin C: 176.12 g/mol; iron: 56 g/mol). The molar ratio of phytic acid to iron was obtained after dividing the moles of phytic acid by the moles of iron. This method is also applied to calculate the ratio of vitamin C to iron [13, 22]. To increase iron absorption, the molar ratio of phytic acid to iron and molar ratio of vitamin C to iron should ideally be ≤1 and 2 : 1, respectively [13, 14].

Venous blood samples (3 mL total) for hematological analyses were drawn from each subject in the morning. The samples were analyzed in the laboratory using a Sysmex-XNL hematology analyzer. The IDA status was measured using four parameters: hemoglobin, mean corpuscular volume (MCV), mean corpuscular hemoglobin (MCH), and mean corpuscular hemoglobin concentration (MCHC). The MCV was used to define red blood cell size, while MCH and MCHC were used to determine the hemoglobin content [23]. Subjects were identified as anemic if their hemoglobin level was below 12 g/dL. IDA was determined by the following criteria: hemoglobin < 12 g/dL, MCV below normal value (<82 fL for age group 11–14 years; <85 fL for age group 15–75 years), MCH < 27 pg, and MCHC < 32 g/dL [24, 25]. Hematocrit was low if the value was ≤36% for children aged between 12 and 14 years and girls aged ≥15 years [26].

All participants obtained written informed consent after they were given a thorough explanation of the study aims, procedures, and associated risks. The study protocol was approved by the Health Research Ethics Committee Faculty of Public Health Diponegoro University (No. 29/EA/KEPK-FKM/2020).

### 2.3. Statistical Analysis

Data were analyzed using the SPSS software version 23. The normality of the data was assessed using the Kolmogorov–Smirnov test, and the Rank Spearman test was used to assess the correlation of variables. The data were considered statistically significant with *p*-value <0.05.

## 3. Results

A total of 176 female students were enrolled in this study. The mean age of the subjects was 15.2 years old. The daily intake of iron was 6.59 mg, and the intake of nonheme iron was higher (5.05 mg) than heme iron (1.52 mg) and iron from meat, fish, and poultry (MFP) (1.16 mg). The molar ratio of phytic acid to iron was 8.72, while the molar ratio of vitamin C to iron was 0.03 (Table 1).

Of the study subjects, 57 had anemia (32.4%) and 39 had IDA (22.2%) (Table 2). The proportion of IDA was higher among students whose parents' education level was higher (50.0% and 27.8% when the mother and father had both attended college and had graduated senior high school, respectively). Those with inadequate nutritional intake had a higher frequency of IDA than those with adequate intake. For hematological parameters, the majority of students with IDA had below normal hematocrit (59.1%), MCH (43.8%), MCHC (78.0%), and MCV statuses (42.6%) (Table 3).

There was a significant correlation between hemoglobin and hematocrit with heme iron and MFP. Non-heme iron, vitamin C, and the molar ratio of phytic acid to iron and vitamin C to iron did not correlate with all hematological parameters (Table 4). Few students had adequate iron intake based on Indonesia RDA, but this was not significantly related to IDA. IDA was also not correlated with drinking tea or drinking tea and/or coffee (Table 5).

## 4. Discussion

In this study, the overall prevalence of anemia (32.4%) was slightly higher than that in the national report, which was 26.8% in children aged 5–14 years and 32.0% in young adults aged 15–24 years [27]. Based on WHO guidelines stating that a prevalence of anemia between 20.0% and 39.9% is of moderate public health significance [28], anemia is indeed a public health problem among the Sundanese adolescent girls in the area. Among all anemic subjects, the proportion of subjects with IDA was 22.2%, which was considerably higher than in other developing countries such as Iran (13.9%) [29], Ethiopia (11%) [6], and Thailand (5.7%) [30]. However, the prevalence of IDA was lower compared to other Asian countries such as Malaysia (34%) [31] and Bangladesh (32%) [32].

Low iron intake might be associated with the high prevalence of IDA observed in the present study. The mean iron intake of the subjects was only 6.59 mg/day, which is lower than the daily intake stated in the Indonesian RDA of 8 mg and 15 mg for females aged 10–12 years and 13–15 years, respectively [33]. Furthermore, the iron intake of the study subjects was mainly from nonheme iron that has lower bioavailability. However, this factor did not significantly affect the iron status in terms of the plant-based Sundanese diet. On the other hand, heme iron and MFP showed a positive correlation with the hematological parameters hemoglobin and hematocrit value. A study in Korea supported this finding in which anemic adolescent girls, as indicated by low hemoglobin concentration, consumed less red meat than those without anemia [34]. Girls who consumed meat (beef, mutton, pork) <4 times/week were more than twice as likely to have iron deficiency compared to those who consumed meat ≥4 times/week [35]. Low hemoglobin concentration was also associated with infrequent consumption of fish and poultry, as well as milk and dairy products [36]. The hematocrit value was also likely to be affected by the socioeconomic status. People with low socioeconomic status tended to have a low hematocrit value, which was related to poor intake and absorption of iron [37]. Moreover, the menu at boarding schools in developing countries generally offers a limited amount of animal-based food and lacks dietary diversity, thus increasing the risk of poor iron status [38].

Not all of the hematological parameters were related to intake of inhibitors and enhancers of iron absorption. The presence of phytic acid and vitamin C in the diet did not significantly affect iron status in the Sundanese girls in this study. This result could be because food sources for vitamin C and iron were not consumed together in the same meal so that vitamin C could not optimally function as an iron absorption enhancer. In addition, the amount of vitamin C consumed by the students (6.34 mg/day) was much lower than the recommended amount of 50 mg/day, 65 mg/day, and 75 mg/day for females aged 10–12 years, 13–15 years, and 16 years and older, respectively. A study in the Philippines showed that vitamin C could affect hemoglobin concentration if the intake exceeded 24 mg/day [39]. The molar ratio of phytate to iron and the molar ratio of vitamin C to iron were used as a determinant of iron absorption, wherein a higher ratio of phytate to iron indicated higher phytate intake and lower iron absorption. On the other hand, a higher molar ratio of vitamin C to iron could increase the absorption of iron and overcome the inhibition caused by phytate intake [40]. In this study, the molar ratio of phytate to iron was high, while the molar ratio of vitamin C to iron were low, indicating iron inhibition. However, the daily intake of phytate was inadequate and vitamin C intake was far lower than recommended. Thus, neither was likely to have had a considerable effect on the iron status.

We also found no significant relationship between consuming tea and consuming tea and/or coffee with IDA. Several studies showed that coffee and tea were not associated with iron deficiency in healthy people with no risk of iron deficiency [41]. Hogenkamp et al. found that iron deficiency and IDA were not significantly explained by black tea consumption in a black adult population in South Africa [42]. Sung et al. stated that green tea intake was not related to serum-ferritin levels, but coffee consumption was associated with lower serum-ferritin levels in Korean adults [43]. Another study found that the mean serum-ferritin concentration was not related to black, green, and herbal tea consumption in men, premenopausal, or postmenopausal women [41]. Evidence suggested that the type of food consumed had a greater influence on iron absorption than the effect of drinking coffee or tea. Coffee and tea were more likely to inhibit the absorption of nonheme iron from plant-based foods but have very little effect on heme iron from animal foods [8]. Similarly, tea consumption in 2,573 French men (*n* = 954) and women (*n* = 1,639) had no influence on iron status [44]. Another cross-sectional study with 157 Indian participants found no differences in anemia prevalence between men and women who consumed diets that contained high and low tannin amounts [41]. Hence, consuming tea and coffee could inhibit iron absorption to cause anemia, but IDA, on the other hand, was influenced by various factors such as the type of food consumed (heme or nonheme iron), when tea and coffee was consumed (preferably 1 hour before eating to not affect iron absorption), and the level consumption of substances that increase iron absorption from food. In the present study, most students only drank coffee and tea occasionally, for instance, after waking up in the morning and during late-night study to help them stay awake, and thus neither was likely to affect iron absorption that leads to IDA.

This study also found no association between red cell indices (MCV, MCH, and MCHC) and all variables analyzed. Nevertheless, MCV and MCH values were somewhat below normal, implying the presence of IDA. Considering that the Sundanese diet is similar to a vegetarian diet, we compared the MCV and MCH values in those two groups. This comparison showed that MCV values in our study were just above that for vegetarians (79.8 fL vs. 78.4 fL), while MCH values were slightly lower (26.0 pg vs. 27.2) [45]. Furthermore, MCV and MCH values in Sundanese girls were similar to those for high school girls who suffer iron deficiency without anemia in Nakhon Si Thammarat, Thailand (MCV = 80.0 fL; MCH = 26.9 pg) [30].

## 5. Conclusions

The prevalence of IDA was high in Sundanese adolescent girls studying at Islamic boarding schools in Indonesia. However, the Sundanese diet, which consists mostly of plant-based foods, was not a factor that caused IDA. Instead, IDA had a greater association with the consumption of less animal-based foods that have higher bioavailability of iron. Hence, to reduce the incidence of IDA, animal-based foods should be offered more frequently at these schools.

## Figures and Tables

**Table 1:** Average daily nutritional intake, molar ratios, and hematological characteristics of adolescent girls (*N* = 176).

Variables	Mean ± SD

Age (years)	15.21 ± 1.76
Nutritional intake	
Energy (kcal/day)	1365.36 ± 580.04
Protein (g/day)	30.22 ± 12.13
Iron (mg/day)	6.59 ± 2.87
Heme-iron (mg/day)	1.52 ± 1.65
Meat, fish, poultry (MFP) (mg/day)	1.16 ± 1.67
Nonheme iron (mg/day)	5.05 ± 2.45
Phytic acid (mg/day)	606.36 ± 274.94
Vitamin C (mg/day)	6.34 ± 12.07
Molar ratio	
Phytic acid:iron	8.72 ± 4.32
Vitamin C:iron	0.03 ± 0.05
Hemoglobin (g/dL)	12.29 ± 1.39
Hematocrit (%)	37.72 ± 3.35
Erythrocyte (10^6^/µL)	4.75 ± 0.41
MCV (fL)	79.83 ± 7.75
MCH (pg)	26.04 ± 3.18
MCHC (g/dL)	32.54 ± 1.36

**Table 2:** Proportion of anemia and iron deficiency anemia (IDA) among adolescent girls (*N* = 176).

Variable	*N* (%)

Hemoglobin status	
Anemia	57 (32.4)
Normal	119 (67.6)
IDA status	
IDA	39 (22.2)
Non-IDA	137 (77.8)

**Table 3:** Sociodemographic characteristics, nutritional intake based on recommended dietary allowance, and the hematological parameter status of adolescent girls with and without iron deficiency anemia (*N* = 176).

Variable	Iron deficiency anemia		Total *N* (%)
	Yes *N* (%)	No *N* (%)	

Education level			
No education	0 (0.0)	10 (100.0)	10 (5.7)
Elementary	1 (7.1)	13 (92.9)	14 (8.0)
Junior high	38 (25.0)	114 (75.0)	152 (86.4)
Father's education level			
No education	0 (0.0)	2 (100.0)	2 (1.1)
Elementary	23 (21.3)	85 (78.7)	108 (61.4)
Junior high	10 (22.2)	35 (77.8)	45 (25.6)
Senior high	4 (23.5)	13 (76.5)	17 (9.7)
College	2 (50.0)	2 (50.0)	4 (2.3)
Mother's education level			
No education	0 (0.0)	2 (100.0)	2 (1.1)
Elementary	23 (22.8)	78 (77.2)	101 (57.4)
Junior high	11 (20.8)	42 (79.2)	53 (30.1)
Senior high	5 (27.8)	13 (72.2)	18 (10.2)
College	0 (0.0)	2 (100.0)	2 (1.1)
Father's employment status			
Unemployed	0 (0.0)	1 (100.0)	1 (0.6)
Employed	39 (22.3)	136 (77.7)	175 (99.4)
Mother's employment status			
Unemployed	35 (23.3)	115 (76.7)	150 (85.2)
Employed	4 (15.4)	22 (84.6)	26 (14.8)
Energy intake			
Inadequate	37 (23.7)	119 (76.3)	156 (88.6)
Adequate	2 (10.0)	18 (90.0)	20 (11.4)
Protein intake			
Inadequate	39 (22.7)	133 (77.3)	172 (97.7)
Adequate	0 (0.0)	4 (100.0)	4 (2.3)
Iron intake			
Inadequate	39 (22.5)	134 (77.5)	173 (98.3)
Adequate	0 (0.0)	3 (100.0)	3 (1.7)
Phytic acid intake			
Inadequate	28 (25.0)	84 (75.0)	112 (63.6)
Adequate	11 (17.2)	53 (82.8)	64 (36.4)
Vitamin C intake			
Inadequate	39 (22.4)	135 (77.6)	174 (98.9)
Adequate	0 (0.0)	2 (100.0)	2 (1.1)
Hematocrit status			
≤36	26 (59.1)	18 (40.9)	44 (25.0)
>36	13 (9.8)	119 (90.2)	132 (75.0)
MCH status			
<27	39 (43.8)	50 (56.2)	89 (50.6)
≥27	0 (0.0)	87 (100.0)	87 (49.4)
MCHC status			
<32	39 (78.0)	11 (22.0)	50 (28.4)
≥32	0 (0.0)	126 (100.0)	126 (71.6)
MCV status			
Low	46 (42.6)	62 (57.4)	108 (61.4)
Normal	0 (0.0)	68 (100.0)	68 (38.6)

**Table 4:** Correlation between nutritional intake and iron status in Sundanese adolescent girls at Islamic boarding schools in Indonesia.

Variable	(*r;p*) value				
	Hemoglobin	Hematocrit	MCV	MCH	MCHC

Heme iron	0.195; 0.009	0.179; 0.018	−0.014; 0.850	0.038; 0.617	0.095; 0.210
MFP	0.195; 0.009	0.190; 0.011	0.003; 0.965;	0.040; 0.601	0.077; 0.308
Nonheme iron	0.075; 0.323	0.086; 0.254	0.103; 0.174	0.073; 0.334	0.032; 0.670
Phytic acid	0.074; 0.331	0.070; 0.358	0.073; 0.338	0.072; 0.344	0.078; 0.305
Vitamin C	0.020; 0.796	0.088; 0.245	−0.013; 0.869	−0.076; 0.315	−0.114; 0.132
Molar ratio of vitamin C:iron	−0.027; 0.724	0.039; 0.603	−0.060; 0.427	−0.118; 0.118	−0.127; 0.094
Molar ratio of phytic acid:iron	−0.116; 0.126	−0.097; 0.199	−0.031; 0.686	−0.057; 0.449	−0.049; 0.519

**Table 5:** Correlation of iron deficiency anemia with drinking tea and coffee.

Variable	Iron deficiency anemia		*p*-value
	Yes (%)	No (%)	

Drinking tea			
Yes	9 (22.0)	32 (78.0)	0.971
No	30 (22.2)	105 (77.8)	
Drinking tea and/or coffee			
Yes	11 (22.4)	38 (77.6)	0.954
No	28 (22.0)	99 (78.0)	

## Data Availability

The data used to support the findings of this study are available from the corresponding author upon request.
